# Genotyping DNA pools on microarrays: Tackling the QTL problem of large samples and large numbers of SNPs

**DOI:** 10.1186/1471-2164-6-52

**Published:** 2005-04-05

**Authors:** Emma Meaburn, Lee M Butcher, Lin Liu, Cathy Fernandes, Valerie Hansen, Ammar Al-Chalabi, Robert Plomin, Ian Craig, Leonard C Schalkwyk

**Affiliations:** 1 Social, Genetic and Developmental Psychiatry Centre, Box Number P082, Institute of Psychiatry, De Crespigny Park, London, SE5 8AF, UK; 2 Department of Neurology, Section of Neurogenetics, Box Number P043, Institute of Psychiatry, De Crespigny Park, London, SE5 8AF, UK

## Abstract

**Background:**

Quantitative trait locus (QTL) theory predicts that genetic influence on complex traits involves multiple genes of small effect size. To detect QTL associations of small effect size, large samples and systematic screens of thousands of DNA markers are required. An efficient solution is to genotype case and control DNA pools using SNP microarrays. We demonstrate that this is practical using DNA pools of 100 individuals.

**Results:**

Using standard microarray protocols for the Affymetrix GeneChip^® ^Mapping 10 K Array Xba 131, we show that relative allele signal (RAS) values provide a quantitative index of allele frequencies in pooled DNA that correlate 0.986 with allele frequencies for 104 SNPs that were genotyped individually for 100 individuals. The sensitivity of the assay was demonstrated empirically in a spiking experiment in which 15% and 20% of one individual's DNA was added to a DNA pool.

**Conclusion:**

We conclude that this approach, which we call SNP-MaP (SNP *m*icroarrays *a*nd *p*ooling), is rapid, cost effective and promises to be a valuable initial screening method in the hunt for QTLs.

## Background

The success of linkage mapping in Mendelian traits led to great optimism that the same approach could be harnessed to identify genes in disorders or traits without clear Mendelian inheritance patterns, so called complex or quantitative traits. In contrast to phenotypes that are controlled by single genes, in complex traits there is no clear-cut mode of transmission. It is likely that quantitative traits are influenced by many genes of small effect size (called quantitative trait loci, QTLs), with factors such as gene-gene interactions (epistasis), pleiotropy, and gene-environment interactions complicating matters further [1,2].

Although many linkage screens for complex disorders have been reported, linkage studies are underpowered to detect genes of small effect size [3]. In contrast, case-control association analysis, even using stringent significance levels, promises to provide the power required to detect QTLs that are too weak to be detected by linkage analysis alone [2]. However, this power comes at an expense, as a systematic genome wide association study requires very large numbers of DNA markers, perhaps as many as 500,000 [4,5]. Furthermore, the ability to detect QTLs of small effect size requires large samples. For example, 80% power (*p *< .01, two tailed) to detect an effect of 0.5% in an unselected sample requires samples of at least 1000 individuals [6].

Consequently, considerable effort has gone into developing high-throughput genotyping methodologies that allow the genotyping of dense marker sets in large sample sizes quickly, accurately, with minimal optimisation and very low unit cost [7]. Until such technologies become widely available, one way to address the cost, time and labour involved in using large sample sizes is to perform analyses not on individual DNA samples, but on pools made up of DNA from multiple individuals for cases and for controls, a technique that can dramatically reduce the genotyping burden [8]. There is a growing literature addressing methodological issues such as DNA pool construction, genotyping assays, and statistical analysis [9-12], and the strengths and weaknesses of DNA pooling have recently been reviewed [13]. DNA pooling has been used successfully to identify replicated associations with complex traits [14-16]. However, using the current SNP genotyping methodologies on DNA pools even for a small number of DNA pools for a dense marker set is still labour-intensive and expensive. One solution to the problem of genotyping many DNA markers is SNP genotyping microarrays which use a one-primer assay to genotype thousands of SNPs, offering the first real hope of a systematic survey of DNA variation in the human genome. However, microarrays can be used only once and are expensive in studies consisting of the large samples needed to detect QTLs of small effect size. One solution to the QTL conundrum is to combine both DNA pooling and SNP microarrays, an approach that we call SNP-MaP (SNP *m*icroarrays *a*nd *p*ooling), which can dramatically reduce the cost of screening large numbers of SNPs on large samples.

We hypothesised that quantitative estimates of allele frequencies – especially the relative allele frequencies comparing groups like cases and controls – can be derived from pooled DNA using SNP genotyping microarrays, similar to the way that expression microarrays estimate quantitative frequencies for mRNA transcripts [17].

Affymetrix software (GDAS) uses the hybridisation fluorescence signals from the SNP microarrays to generate 'Relative Allele Signals' (RAS), a ratio of the measurement of allele A to the summed measurement of alleles A and B. Thus, RAS values vary between 0.0 and 1.0. Two independent RAS values are derived for each SNP from the sense strand (RAS1) and the anti-sense strand (RAS2). As explained in the Methods, Affymetrix software plots RAS1 scores against RAS2 scores and uses empirically derived clustering information to trichotomise these RAS scores as genotypes for DNA of an individual. RAS values near 0.0 are identified as a BB homozygote, 0.5 as an AB heterozygote, and 1.0 as an AA homozygote. We propose that RAS values can be used as quantitative indexes of allele frequencies in DNA pools.

The purpose of our current report is to follow up our previous study that addressed the feasibility of SNP-MaP [18]. We explore the reliability validity and sensitivity of SNP-MaP in greater detail using Affymetrix GeneChip^® ^Mapping 10 K Array Xba 131 which genotypes more than 10,000 SNPs.

We constructed a control DNA pool consisting of 100 individuals independently three times (control pool A, B, and C), each assayed on triplicate microarrays. We used these replicate control pools to assess the reliability of estimating allele frequencies from pooled DNA. To assess validity and sensitivity, we compared allele frequency estimates from microarray assays using pooled DNA to individual genotyping. In addition, in order to assess sensitivity experimentally, we reconstructed two 'case' DNA pools that differed by 15% and 20% in allele frequencies from the controls by spiking an aliquot of a control pool with an individual's DNA who was also individually genotyped on the microarray. Each case pool was assayed on duplicate microarrays.

## Results

### Validation of pool construction

Before using the microarrays, we genotyped the 9 DNA pools for 3 SNPs using SNaPshot™ ddNTP primer extension reaction kit (Applied Biosystems^©^) in order to confirm that the control DNA pools A, B and C were representative of the 100 individuals used to construct the pools. The results indicated that pool construction was valid in that, for the three SNPs, the pools yielded similar allele frequency estimates. Most importantly, these estimates, and especially their averages across the pools, are highly similar to the allele frequencies based on individual genotyping. For example, the average pooled allele frequencies (corrected for *k *see below and Methods) and individual genotyping allele frequencies, respectively, were 0.914 and 0.918 for rs1003063, 0.438 and 0.464 for rs15643193, and 0.639 and 0.634 for rs956122. SNaPshot™ has previously been shown to estimate pool allele frequencies with a high degree of accuracy if *k *is applied [12].

### Summary of 10 K GeneChip results for pooled DNA

We consistently obtained good signal detection rates across all 14 microarrays used in the study, with an average signal detection of 83.9% (range of 60.54% to 98.59%) using the default analysis parameters. SNPs were excluded if there was inadequate discrimination between specific versus non-specific hybridisation of DNA to the probes.

We obtained RAS1 and RAS2 values for 6077 SNPs across all 9 microarrays for the control pools A, B and C. For the spiked pools, RAS1 and RAS2 values were obtained for 9908 SNPs for the 15% spiked pool and 6668 SNPs for the 20% spiked pools.

### Validation: comparing allele frequencies from pooled DNA and population estimates

We have previously shown that an average of RAS1 and RAS2 values (RAS_av_) estimate pooled allele frequencies more accurately then RAS1 or RAS2 alone [18].

RAS_av _values were calculated for each SNP on the 3 microarrays for each of the 3 DNA control pools. These RAS_av _values were then averaged across the 9 microarrays. The resulting allelic frequency estimates from pooled DNA were correlated with SNP allele frequencies determined by individually genotyping for an independent Caucasian sample. The latter data are publicly available from the NetAffx™ Analysis Centre , a web-based tool providing extensive annotation for each SNP on the 10 k microarray derived from Affymetrix internal validation studies.

**Figure 1:**
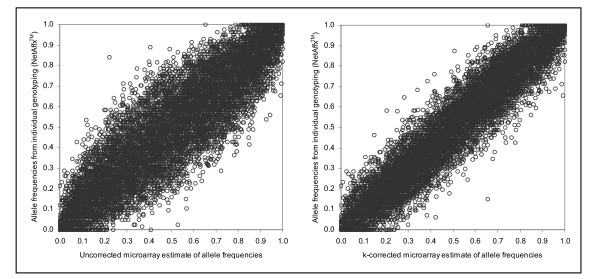
**a**: RAS_av _values derived from between 1–9 replicate assays of the control pool DNA, compared to published population allele frequency estimates for the entire array. *N *= 11,533. **1b**: *k*-corrected RAS_av _values derived from between 1–9 replicate assays of the control pool, compared to published population allele frequency estimates for the entire array (*N *= 11,533).

Figure 1a shows a scatterplot between the RAS_av _estimates of allele frequencies for pooled DNA versus the allele frequency estimates from individual genotyping for 11,533 SNPs. The correlation between the pooled DNA and individual genotyping estimates is 0.901, indicating that RAS values can be used to provide a valid quantitative measure of allele frequency in pooled DNA. Nonetheless, the absolute mean difference between microarray estimates of pooled allele frequencies and NetAffx™ population allele frequencies was 0.094 but varies widely (Min 0.00, Max 0.619).

In Figure 1a, the allele frequency estimates from pooled DNA were not corrected for unequal amplification of the two alleles that can occur with pooled DNA. That is, when two alleles are present in equal amounts (as in heterozygous individuals), the allele measurements obtained should be of equal size or intensity. However, for to various reasons [19] equal representation of alleles may not occur.

This bias is corrected by applying a factor, *k *[20], where *k *= A/B, using the equation: A = A/(A+*k*B), where A and B are the measurements of the A and B alleles. *k *corrections for the SNPs on the Affymetrix 10 K microarray were available from a panel of 33 Caucasian individuals assayed on separate microarrays and applied to each SNP (see Methods).

The range of *k *values is very tight, with only a handful of outliers considering the enormous number of SNPs under investigation. 6,813 of 10,084 SNPs have *k *values ranging from 0.5 to 1.5 (we would expect *k *to be 1 if both alleles were being measured equally). The averaged *k *over 10,088 SNPs is 1.14, median = 0.94, max = 93, min = 0.0. It should be noted that in relation to a case-control study design, unless *k *is extreme and allele frequencies rare, *k *correction would not significantly impact on *relative *allele frequency differences between cases and controls, although *absolute *allele frequency estimates would alter. In other words, although *k *correction is needed to compare allelic frequency estimates from pooled DNA and individual genotyping, it is not needed for comparisons between cases and controls.

Figure 1b shows the tighter scatter around the line of best fit when the pooled estimates are *k-*corrected. The correlation between the pooled and individual genotyping estimates increased from 0.901 to 0.953. The mean difference between *k*-corrected microarray estimates and NetAffx™ is also attenuated from .094 to .064.

### Validation: comparing allele frequencies for pooled DNA and individual genotyping

**Figure 2:**
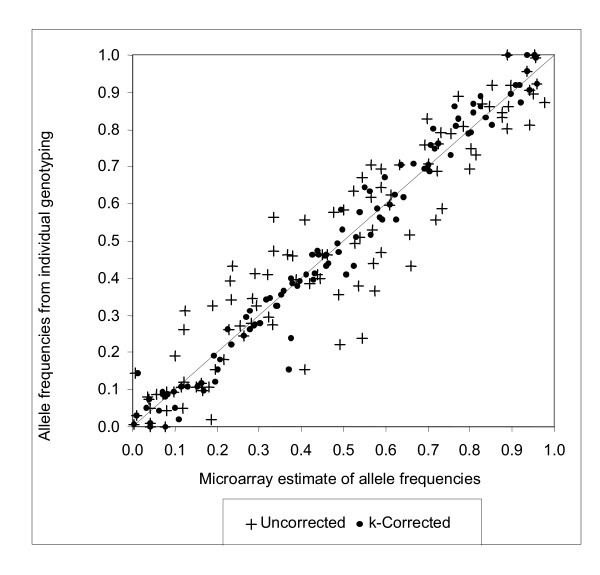
*k*-corrected and uncorrected RAS_av _values derived from nine replicate control pool assays compared to individual genotyping data for 104 SNPs.

In addition to using the NetAffx™ population data, we individually genotyped the 100 individuals used to construct the control pool for 104 SNPs. The results are shown in Figure 2. As expected, the 104 SNP allele frequencies generated by the micorarrays are highly correlated (0.924) with the NetAffx™ population data, and correlate even better with the individual genotyping data for individuals in the pool (0.942). The mean difference between microarray estimates and individual genotyping was .077. When *k *correction was applied, the correlation between *k-*corrected microarray estimates and individual genotyping increased to 0.986, and the mean difference between *k-*corrected microarray estimates was attenuated from .077 to .036.

### Estimation of experimental errors

**Figure 3:**
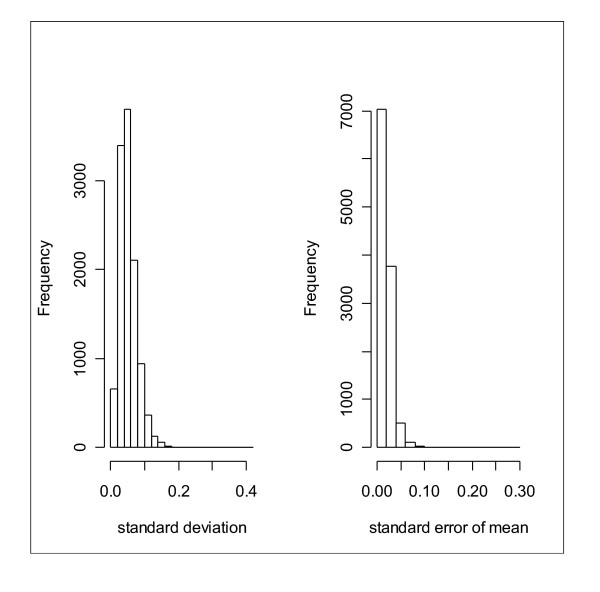
Distribution of standard deviation and standard error in RAS_av _across all nine replicate control pool assays.

The previous analyses are based on RAS_av _values averaged across the 9 microarrays. In order to assess the technical reliability of deriving allele frequency estimates from the DNA pools, we compared RAS_av _for the three technical replicates within each DNA pool and between the three DNA pools. (See Figure 3.) As expected given the high correlation between pooled estimates and individual genotyping, correlations of RAS_av _values within pool triplicates are very high (0.945 to 0.968), as are correlations between pools (0.94 to 0.982). The dispersion of the RAS_av _values cross the nine control microarrays is encouragingly narrow: all but 19 SEMs are under 0.1 and 8,695 are 0.025 or less.

For those SNPs where we have RAS values across all 9 pools (*n *= 6077), the median standard deviation is .042. Using this figure to estimate the power of a hypothetical experiment where we have the same number of case pools, for a .05 significance level we would have 80% power to detect an allelic frequency difference of 0.05, and virtual certainty of detecting an allelic frequency difference of 0.10.

By fitting a random intercept model for each SNP to the 9 RAS_av _values, it is possible to estimate the variance components attributable to pool construction (τ) and the measurement error (σ). σ and τ are assumed to be independently normally distributed. The distribution of  is quite similar to the overall standard deviation (see figure 3) because tau is, as expected, quite small, ranging from 9 × 10^-7 ^to 2 × 10^-4^, with a median of 3 × 10^-5^. An estimate of 1 × 10^-4 ^has been made from comparable pools using different methods [21].

### Sensitivity

The sensitivity of SNP-MaP was assessed empirically in a spiking experiment in which 15% and 20% of one individual's DNA was added to control pool D to create two new pools analogous to 'case' pools. Each of the spiked pools was assessed on duplicate microarrays. The individual's DNA was also genotyped on a microarray in order to calculate the allelic frequencies of the spiked pools.

Based on the power calculations presented above, in this spiking experiment we would expect to have roughly 52% power to detect allelic frequency differences of 0.10 between the control and spiked pools. This is less than the power estimate from the control DNA pools because here we have only two replicates of each spiked pool, and nine replicates of the control pool, which is equivalent to having equal groups of *n *= 3.27 (the harmonic mean of 9 and 2). The expected differences in the spiked pool are dependent upon the original allele frequencies in the control pool because we used a fixed total amount of (i.e. the amount of pool DNA is reduced to make room for the spike DNA). For the SNPs where the expected allele frequency difference in the 15% and 20% spiked pools was more than 0.10 and we had complete data (*n *= 658), a significant *t*-test difference was observed for 379 SNPs (58%), supporting our predicted power calculations. As shown in Table 2, the observed and predicted differences between spiked ('case') and control correspond well on average over the entire frequency range, although the predicted allele frequency differences are greater than the observed differences. The expected allele frequencies are calculated from frequencies measured in the control pools, and are greatest for low frequency alleles, some of which appear low by chance due to measurement error. The apparent bias shown in table 2 is thus due to regression to the mean.

## Discussion

We have demonstrated that the SNP-MaP method can tackle the QTL problem of large samples and large numbers of SNPs. Specifically, allele frequencies can be accurately estimated from pooled DNA allelotyped on microarrays using the sophisticated allele-specific hybridisation method packaged in the commercially available Affymetrix GeneChip^® ^system. The validity and sensitivity of the SNP-MaP method was shown by the correlation of 0.986 between estimates of allele frequency from pooled DNA and individual genotyping. The sensitivity 'spiking' experiment confirmed these estimates, suggesting that case-control pool allele frequency differences on the order of 0.10 could be detected above the noise of measurement error using this method. With this level of sensitivity, the SNP-MaP approach should be able to detect QTLs of modest effect size if large samples are used. The benefit of DNA pooling is that large samples cost no more to genotype than small samples.

These results make it reasonable to proceed to case-control studies of complex traits. For example, we are using the SNP-MaP method in a large-scale study of children of low versus high reading ability. In an actual association study, sampling variation is added to the parameters considered in the present study which focused on technical replicates within and between DNA pools of the same group of individuals and compared allele frequency estimates for pooled DNA from this group of individuals to individual genotyping. However, in an actual association study, pooled DNA estimates are not compared within and between pools of the same individuals but rather between different pools consisting of different individuals. For this reason, in an actual association study, sampling variation would be considered and could be estimated parametrically by including in the design sub-pools of independent samples of cases and controls. This would permit the use of standard parametric statistics comparing the mean allelic frequencies of cases and controls in which *N *is the number of subpools. In addition, using at least two microarrays on each subpool would alleviate technical variation between microarrays and sub-pools. It should be emphasised that DNA pooling is best construed as nominating SNPs that are then confirmed with individual genotyping.

We have used the quantitative RAS values, designed to produce a robust allele call when assessed for individuals, to estimate allele frequencies for pooled DNA. It is likely that a more elegant way can be found to derive an allele frequency estimate from the 40 oligonucleotide probes present for each SNP (20 matches and 20 mis-matches). For example, it has been reported in a study of gene expression differences that increased power can be obtained by testing for differences for each probe rather than for differences averaged over the entire probe set level [22].

The tests used here were not corrected for multiple testing because the intent is to use this method not as a definitive analysis, but rather as a rapid initial screen to reduce the number of candidate SNPs to be submitted to individual genotyping for the large samples needed to detect QTLs of small effect size. With 10,000 SNPs, a large number of false positive results are expected by chance and so we recommend using a multi-stage replication design in order to balance false positives and false negatives in the selection of SNPs for individual genotyping [13].

Our analyses of SNP-MaP were based on the 10,000 SNP microarray, but should also be applicable to the 100 k Affymetrix GeneChip^® ^system launched in June 2004 and the 500 k Affymetrix GeneChip^® ^system scheduled for the end of 2005. Although the SNPs on the Affymetrix arrays are chosen on the basis of coverage of the whole genome (not on the basis of functionality or location in or near coding regions), 500,000 SNPS approaches a comprehensive genome scan for association based on linkage disequilibrium. The SNP-MaP approach will also be relevant to other types of SNP microarrays such as a microarray that emerges from the exon re-sequencing project at the Sanger Centre which aims to identify all nonsynonymous (nsSNPs), which are likely to be functional because the polymorphism involves an amino acid difference in translation. Microarrays with functional SNPs will facilitate direct association analyses with increased power as compared to indirect association analyses based on linkage disequilibrium [23].

## Conclusion

In summary, we have shown that the SNP-MaP method is a reliable and powerful approach to screen thousands of loci using large sample sizes. Employing case-control designs using SNP-MaP will speed-up the SNP nomination process for individual genotyping, and thus help detect SNPs of small effect size more efficiently.

## Methods

### Samples

The sample consisted of 100 Caucasian individuals (51 females and 49 males) randomly selected from an ongoing study of cognitive ability that is described elsewhere [24].

### DNA quantification and pool construction

DNA samples were quantified using a spectrophotometer (260 nm), and diluted in Te buffer (0.1 mM EDTA, 10 mM Tris HCL, pH 8.0) to a target concentration of 100 ng/μl, before being quantified using fluorimetry (Picogreen^® ^dsDNA quantitation reagent Cambridge Bioscience, U.K) and diluted further to a target concentration of 50 ng/μl (± 5%). Pools were constructed independently in triplicate by combining 50 ng of DNA from each individual, producing a final pool concentration of 35.5 ng/μl.

An aliquot of control pool D was reconstructed to form artificial 'case' pools by replacing 15 and 20% of the pool sample with the same quantity of DNA of one genotyped individual (the spike). This changes the quantities of alleles present in a way that depends on their frequency in the pool and the genotype of the spike.

### Validation of pool construction

Three SNPs chosen from the microarray set were genotyped independently in duplicate on the three replicate control DNA pools, A, B and C using SNaPshot™, a commercially available dideoxynucleotide primer extension kit (PE applied biosystems). Electrophoresis of the samples was performed on an ABI Prism^® ^3100 genetic analyser, analysed with Genotyper^® ^3.7, and the allele frequencies derived from peak heights.

The correction factor, *k*, was calculated by individually genotyping seven known heterozygous individuals using the SNaPshot™ method for each of the three SNPs. The pooled allele frequency estimates were then corrected using the equation A = A/(A+*k*B), where A and B are the peak heights of alleles A and B respectively.

### Individual genotyping

104 randomly chosen SNPs from the microarray were genotyped in the 100 individuals that were used to construct the DNA control pool. The individual genotyping was outsourced to Kbiosciences, who use a mixture of competitive allele specific PCR (KASPar) and TaqMan genotyping assays .

### Genotyping DNA pools on the microarray

The individual spike DNA and 13 pooled DNA samples were genotyped using Affymetrix GeneChip^® ^Mapping 10 K Array Xba 131. Standard procedures and default analysis parameters for individual DNA samples were employed, and each assay was independently amplified before hybridization. The scans were performed using GCOS V1.0 and the images (cel.files) were analyzed using GDAS V2.0.

### Derivation of allele frequencies from pooled DNA

The Affymetrix microarray uses a probe quartet as the basic unit for detecting different genotypes with a DNA sample. Each probe quartet consists of a perfect match (PM) and a mis-match (MM) probe for alleles A and B on both the sense and anti-sense strands. To make the genotyping more reliable, 7 probe quartets are used, with the polymorphic nucleotide having different shifts from the center of the 25-mer probe sequence. The best five quartets are used to create 40 hybridization intensity values.

A detection filter automatically blocks weak or unreliable signals by comparing the discrimination between perfect match and mis-match cells using the formula (PM-MM)/(PM + MM). Feature extraction processes the intensity values for all SNPs that pass the pre-determined discrimination threshold.

In feature extraction, PM probes for the sense strand of allele A (PM_A_) are corrected for the noise of non-specific hybridisation by averaging the mis-match values of both alleles (A and B) on the sense strand [(MM_A _+ MM_B_)/2] and subtracting this from the PM_A _value. This procedure for correcting the sense strand of allele A is repeated for the anti-sense strand of allele A, the sense strand of allele B and the anti-sense strand of allele B, resulting in four relative intensity values.

Relative allele signals (RAS) are then calculated for the sense (RAS1) and anti-sense strands (RAS2) using the formula [PM_A_/ (PM_A _+ PM_B_)]. This is done for each of the five quartets for each SNP and the median RAS1 and median RAS2 value is used to identify genotypes for individual DNA [25]. Plotting RAS1 scores against RAS2 scores, a clustering algorithm is used to determine individual genotypes. That is, if the two RAS values cluster near 0.0, they are identified as a BB homozygote; 0.5 for an AB heterozygote, and 1.0 for an AA homozygote. Affymetrix software (GDAS) incorporates empirically derived boundaries for each genotype's cluster, which is used to indicate genotypes; RAS scores falling outside these cluster boundaries are not assigned genotypes. For pooled DNA, we used only the detection filter, feature extraction processes, and RAS scores, disregarding the automated genotype calls. We used the average of the sense (RAS1) and anti-sense RAS2; RAS_av _as an estimate of allele frequency in the DNA pools.

As mentioned, a correction factor, *k*, is used to improve the accuracy of allele frequency estimates from pooled DNA. *k *was empirically derived from a panel of 33 individuals assayed on microarrays. *k *was calculated for each strand by dividing the mean of allele A (RAS1 or RAS2) by the mean of allele B (1 minus allele A). The mean of RAS1 and RAS2 was calculated by averaging a set of RAS1 and RAS2 scores from a panel of between 1 and 33 heterozygous individuals. In total, we obtained an estimate of *k *for 10,084 SNPs. Derivation and implementation of *k *in correcting pooled DNA estimates of allele frequency using microarrays are described in detail elsewhere [26].

## Authors' contributions

EM and LB constructed the DNA pools, performed the microarray assays (with assistance from LL and CF), statistical analysis (with LS), and drafted the manuscript (with LS). AC and VH performed the assays used to estimate k. RP, IC, LS and CF conceived and designed the study. All authors read and approved the final manuscript.

**Table 1:** Predicted and observed allele frequency differences. The predicted allele frequency differences are the differences we would expect to see based on the allele frequency estimates from the control pools and the genotype of the spiker. The observed allele frequency differences are the actual frequencies observed in the spiked pool as compared to the control pools. Number of SNPs range between 459 and 2621.

Predicted	Observed	N of observations
0.001	0.001	1116
0.010	0.007	2621
0.020	0.017	2478
0.030	0.024	2309
0.040	0.033	2043
0.050	0.042	1789
0.060	0.051	1450
0.070	0.062	1113
0.080	0.069	835
0.090	0.076	595
0.099	0.081	459

## References

[B1] Lander E, Schork NJ (1994). Genetic dissection of complex traits. Science.

[B2] Risch NJ (2000). Searching for genetic determinants in the new millennium. Nature.

[B3] Zondervan KT, Cardon LR (2004). The complex interplay among factors that influence allelic association. Nat Rev Genet.

[B4] Kruglyak L (1999). Prospects for whole-genome linkage disequilibrium mapping of common disease genes. Nat Genet.

[B5] Abecasis GR, Noguchi E, Heinzmann A, Traherne JA, Bhattacharyya S, Leaves NI, Anderson GG, Zhang Y, Lench NJ, Carey A, Cardon LR, Moffatt MF, Cookson WO (2001). Extent and distribution of linkage disequilibrium in three genomic regions. Am J Hum Gen.

[B6] Cardon LR, Bell JI (2001). Association study designs for complex diseases. Nat Rev Genet.

[B7] Syvanen AC (2001). Accessing genetic variation: Genotyping single nucleotide polymorphisms. Nat Rev Genet.

[B8] Daniels J, Holmans P, Williams N, Turic D, McGuffin P, Plomin R, Owen MJ (1998). A simple method for analyzing microsatellite allele image patterns generated from DNA pools and its application to allelic association studies. Am J Hum Genet.

[B9] Curran S, Hill L, O'Grady G, Turic D, Asherson P, Taylor E, Sham P, Craig I, Vaughan P (2002). Validation of single nucleotide polymorphism quantification in pooled DNA samples with SNaPIT. A glycosylase-mediated methods for polymorphism detection method. Molecular Biotechnology.

[B10] Jawaid A, Bader JS, Purcell S, Cherny SS, Sham PC (2002). Optimal selection strategies for QTL mapping using pooled DNA samples. Eur J Hum Genet.

[B11] Le Hellard S, Ballereau SJ, Visscher PM, Torrance HS, Pinson J, Morris SW, Thomson ML, Semple CA, Muir WJ, Blackwood DH, Porteous DJ, Evans KL (2002). SNP genotyping on pooled DNAs: comparison of genotyping technologies and a semi automated method for data storage and analysis. Nucleic Acids Res.

[B12] Norton N, Williams NM, Williams HJ, Spurlock G, Kirov G, Morris DW, Hoogendoorn B, Owen MJ, O'Donovan MC (2002). Universal, robust, highly quantitative SNP allele frequency measurement in DNA pools. Hum Genet.

[B13] Sham PC, Bader JS, Craig I, O'Donovan M, Owen M (2002). DNA pooling: A tool for large-scale association studies. Nat Rev Genet.

[B14] Shifman S, Bronstein M, Sternfeld M, Pisante-Shalom A, Lev-Lehman E, Weizman A, Reznik I, Spivak B, Grisaru N, Karp L, Schiffer R, Kotler M, Strous RD, Swartz-Vanetik M, Knobler HY, Shinar E, Beckmann JS, Yakir B, Risch N, Zak NB, Darvasi A (2002). A highly significant association between a COMT haplotype and schizophrenia. Am J Hum Genet.

[B15] Butcher LM, Meaburn E, Dale PS, Sham P, Schalkwyk LC, Craig IW, Plomin R (2004). Association analysis of mild mental impairment using DNA pooling to screen 432 brain-expressed single-nucleotide polymorphisms. Mol Psychiatry.

[B16] Cope N, Harold D, Hill G, Moskvina V, Stevenson J, Holmans P, Owen MJ, O'donovan MC, Williams J (2005). Strong Evidence That KIAA0319 on Chromosome 6p Is a Susceptibility Gene for Developmental Dyslexia. Am J Hum Genet.

[B17] Zeng W, Chen G, Kajigaya S, Nunez O, Charrow A, Billings EM, Young NS (2004). Gene expression profiling in CD34 cells to identify differences between aplastic anemia patients and healthy volunteers. Blood.

[B18] Butcher LM, Meaburn E, Liu L, Hill L, Al-Chalabi A, Plomin R, Schalkwyk L, Craig IW (2004). Genotyping pooled DNA on microarrays:  A systematic genome screen of thousands of SNPs in large samples to detect QTLs for complex traits. Behav Genet.

[B19] Norton N, Williams NM, O'Donovan MC, Owen MJ (2004). DNA pooling as a tool for large-scale association studies in complex traits. Ann Med.

[B20] Hoogendoorn B, Norton N, Kirov G, Williams N, Hamshere ML, Spurlock G, Austin J, Stephens MK, Buckland PR, Owen MJ, O'Donovan MC (2000). Cheap, accurate and rapid allele frequency estimation of single nucleotide polymorphisms by primer extension and DHPLC in DNA pools.. Hum Genet.

[B21] Barratt BJ, Payne F, Rance HE, Nutland S, Todd JA, Clayton DG (2002). Identification of the sources of error in allele frequency estimations from pooled DNA indicates an optimal experimental design. Ann Hum Genet.

[B22] Barrera L, Benner C, Tao YC, Winzeler E, Zhou Y (2004). Leveraging two-way probe-level block design for identifying differential gene expression with high-density oligonucleotide arrays. BMC Bioinformatics.

[B23] Carlson CS, Eberle MA, Kruglyak L, Nickerson DA (2004). Mapping complex disease loci in whole-genome association studies. Nature.

[B24] Plomin R, Hill L, Craig I, McGuffin P, Purcell S, Sham P, Lubinski D, Thompson L, Fisher PJ, Turic D, Owen MJ (2001). A genome-wide scan of 1842 DNA markers for allelic associations with general cognitive ability: A five-stage design using DNA pooling and extreme selected groups. Behav Genet.

[B25] Liu WM, Di X, Yang G, Matsuzaki H, Huang J, Mei R, Ryder TB, Webster TA, Dong S, Liu G, Jones KW, Kennedy GC, Kulp D (2003). Algorithms for large-scale genotyping microarrays. Bioinformatics.

[B26] Simpson CL, Knight J, Butcher LM, Hansen VK, Meaburn E, Schalkwyk LC, Craig IW, Powell JF, Sham PC, Al Chalabi A (2005). A central resource for accurate allele frequency estimation from pooled DNA genotyped on DNA microarrays. Nucleic Acids Res.

